# A 5-year follow-up of Girdlestone resection arthroplasty in hip infection continued by Total hip replacement: A case report^☆^

**DOI:** 10.1016/j.ijscr.2022.106861

**Published:** 2022-02-25

**Authors:** Iman Solichin, Guntur Utama Putera, Mohamad Walid Kuncoro

**Affiliations:** a Department of Orthopaedic and Traumatology, Faculty of Medicine Universitas Indonesia, Orthopaedic Hospital Purwokerto, Indonesia; b Department of Orthopaedic and Traumatology, Faculty of Medicine Universitas Indonesia, Dr. Cipto Mangunkusumo National Central General Hospital, Indonesia

**Keywords:** Hip infections, Girdlestone, Total hip replacement, Case report

## Abstract

**Introduction:**

Resection arthroplasty of the hip was already performed for recent 100 years. This kind of surgery has been used for a wide variety of hip problems such as infection, degenerative osteoarthritis and prosthetic joint infection. Total hip replacement is remarkable procedure in order to relieve pain and restore hip function. We present this report to describe our experience in treating hip infection by used two stages of surgery.

**Case presentation:**

A 61-years old male suffered left hip infection that leads severe destruction on his joint. He felt badly pain on his hip with significant disability associated it such as limping, restriction of movements, and shortening of the limb. The hip movements were painful and caused walking in antalgic gait. Radiologic examination at that time revealed gross destruction of femoral head as well as acetabulum, irregular and hazy joint margins with diminished joint space. Patient was diagnosed with chronic non-specific inflammation of the hip joint.

**Result:**

Two stages surgery were performed on this patient. Resection arthroplasty firstly performed in order to eradicate the infection completely and alleviate very bothersome pain for improvement of patient's quality of life. Two years later, after patient was ready for next stage mentally, cemented total hip replacement performed to achieve normal function of the hip. Five-years follow up functional outcome was performed. Patient was very satisfied with the result with Harris hip scores was 95.

**Conclusion:**

Resection arthroplasty of the hip followed by a conversion to total hip replacement in hip infection case provide complete infection eradication, good functional outcome and satisfaction for the patient. Although the procedure was time-consuming it can be a choice if the eradication of infection still in a doubt.

## 1 Introduction

Resection arthroplasty of the hip was already performed for recent 100 years [1]. This kind of surgery was an operative procedure that can be a valuable tool to address complex hip problems [1]. Girdlestone resection arthroplasty (Girdlestone procedure) was a common option especially before the introduction of antibiotics and arthroplasty management for infectious or post-traumatic lesion of the hip were limited. When surgery was possible, the option was to resected the femoral head to relieved the painful infected joint [2].

Resection arthroplasty of the hip firstly described by Gathrone Robert Girdlestone in 1928. An English orthopaedic surgeon described resection arthroplasty of the hip as a salvage technique in treating septic arthritis. The clinical situation resulted later was eponymously named as “Girdlestone situation”. In 1928–1950, this procedure was initially indicated for treating hip pyogenic infection. The infections could be caused by hematogenous spread, open fractures, or gunshot wounds. Nowadays the term of Girdlestone resection arthroplasty closely related to management of prosthetic joint infection (PJI) with complete removal of hip prosthesis and leaving the crude articulation between acetabulum and femur. This procedure mostly is a part of two stage surgery in revision hip arthroplasty in the management of PJI [2].

Total hip replacement (THR) is remarkable procedure in order to relieve pain and restore hip function. This procedure was initially developed by Sir John Charnley, an English orthopaedic surgeon, and gained success in the early 1960s. THR provides patients a stable joint with painless and normal gait. Some considerations of contraindication for THR are because of risk of infection or reactivation of the infection. There are others recommendation to deal with long interval between management of active infection and THR procedure [3].

The treatment of advanced hip infection with destruction of joint structure is still controversial. Some options for this condition are arthrodesis, resection arthroplasty and total hip replacement (THR). Resection arthroplasty provides painless hip with a consequence of instability with abnormal gait and shortening. In some reports, THR conversion after resection arthroplasty is a complex procedure and may be less satisfactory [4]. In this report we try to describe our experience in treating hip infection by used two stages of surgery. This case report has been reported in line with the SCARE criteria [5].

## 2 Case presentation

**Fig. 1:**
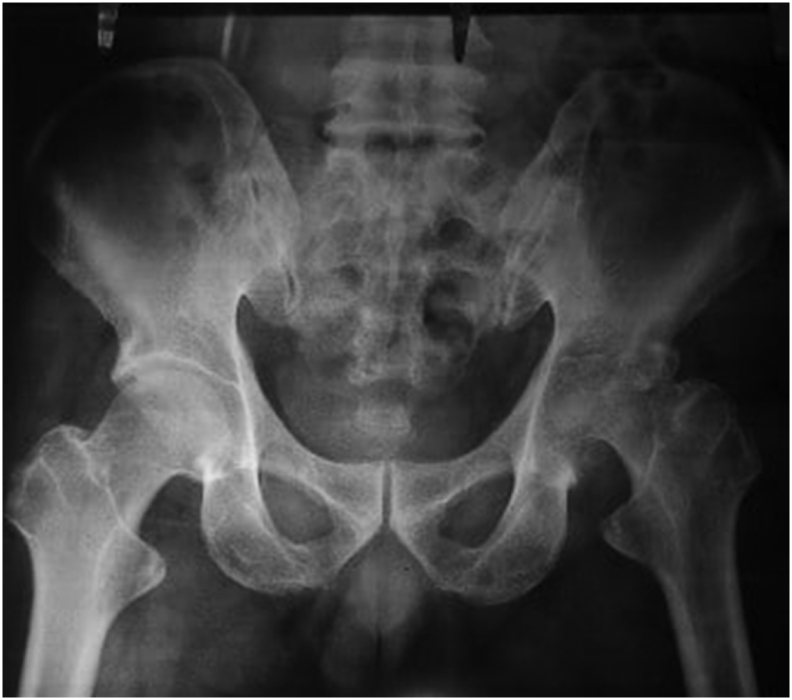
Preoperative x-ray.

A 61-years old male suffered left hip infection that leads severe destruction on his joint. He felt badly pain on his left hip for three months and already sought to many doctors but treated as back problem. There were significant disabilities associated it such as limping, restriction of movements, and shortening of the limb. The hip movements were painful and caused walking in antalgic gait. There were no history of previous fever nor sinuses discharging from the hip region. Radiologic examination at that time revealed gross destruction of femoral head as well as acetabulum, irregular and hazy joint margins with diminished joint space (Fig. 1).

Two stages surgery were performed on this patient. Resection arthroplasty firstly performed in order to eradicate the infection completely and alleviate very bothersome pain for improvement of patient's quality of life. Girdlestone resection of proximal femur was performed with posterior approach, intraoperatively there were swollen, necrotic, and granulation tissue around the joint. Debridement of the necrotic tissue as well as biopsy was performed to eradicate infection locally and further diagnosis respectively. Pathologic anatomy examination revealed a connective tissue fibrosis, fat and some fragments of hyperemic swollen bone contended with PMN leukocyte and lymphocyte. This condition refers to chronic non-specific inflammation of the hip joint.

**Fig. 2:**
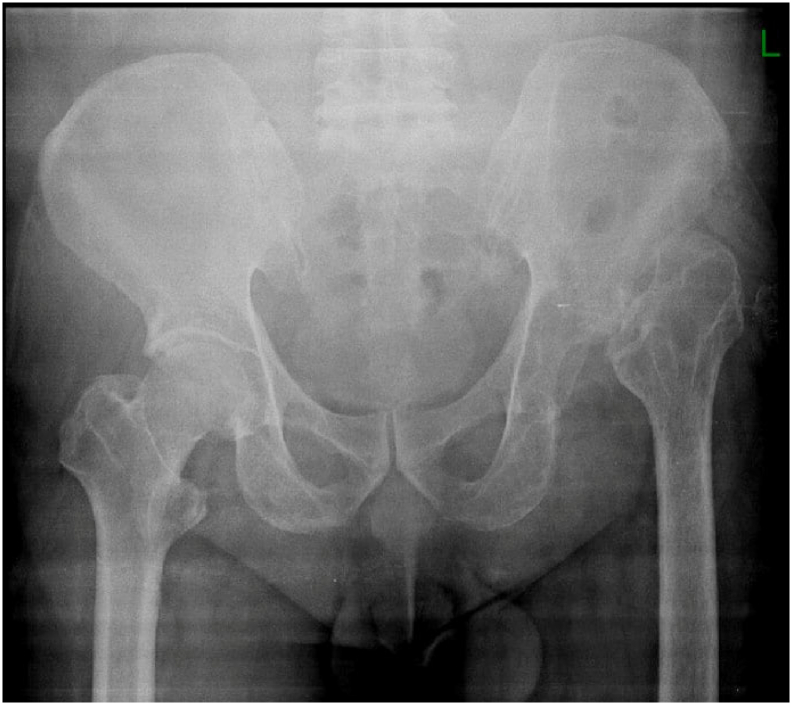
Postoperative x-ray.

Although this first stage surgery result in approximately 5 cm leg length discrepancy (Fig. 2), patient was quite satisfied with the results as pain was reduced, and was able to walk with a cane. Two years after first operation, once the patient was mentally ready for the next operation, cemented total hip replacement was performed. This procedure corrected the discrepancy and patient was able to walk immediately. Postoperative rehabilitation was performed to achieve normal gait and hip joint range of motion.

**Fig. 3:**
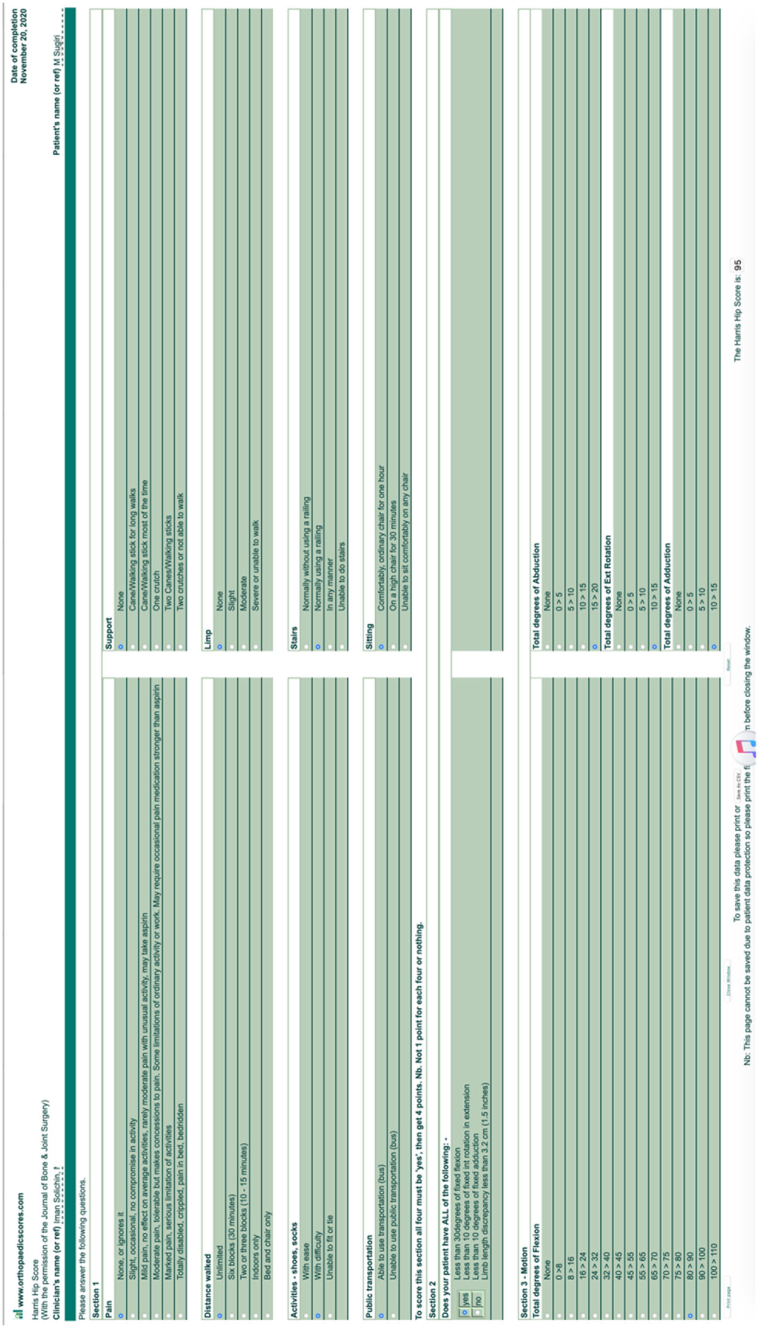
Harris Hip score measurement.

**Fig. 4:**
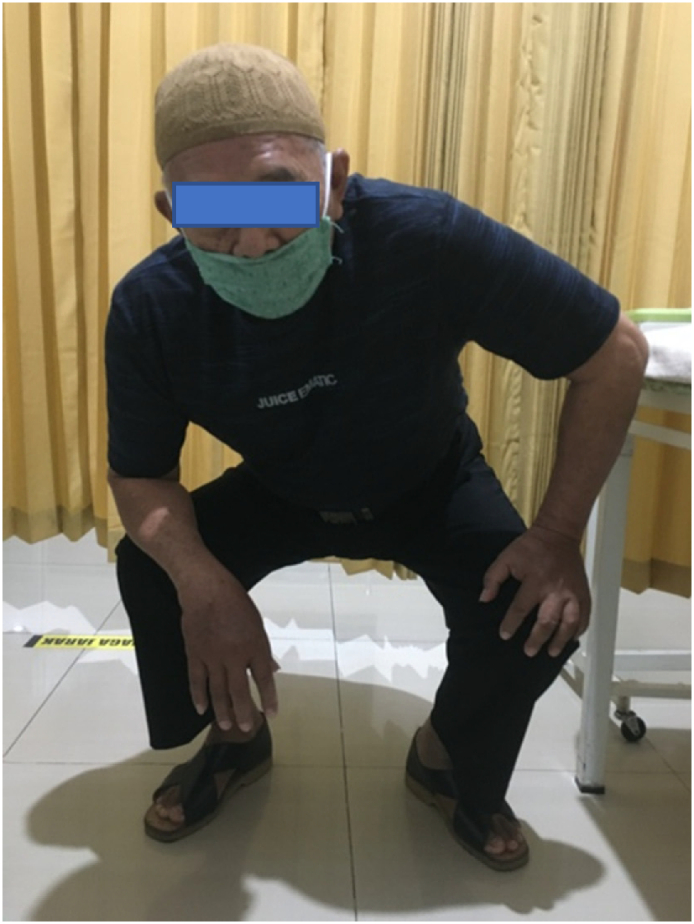
Current clinical condition (squating).

**Fig. 5:**
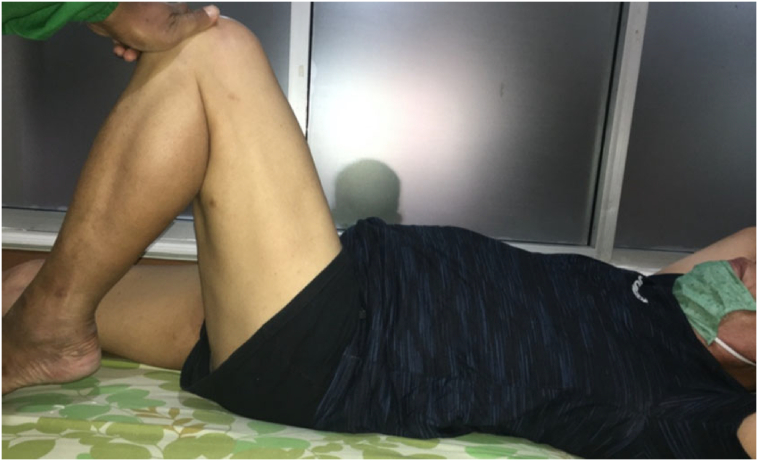
Current clinical condition (hip flexion).

**Fig. 6:**
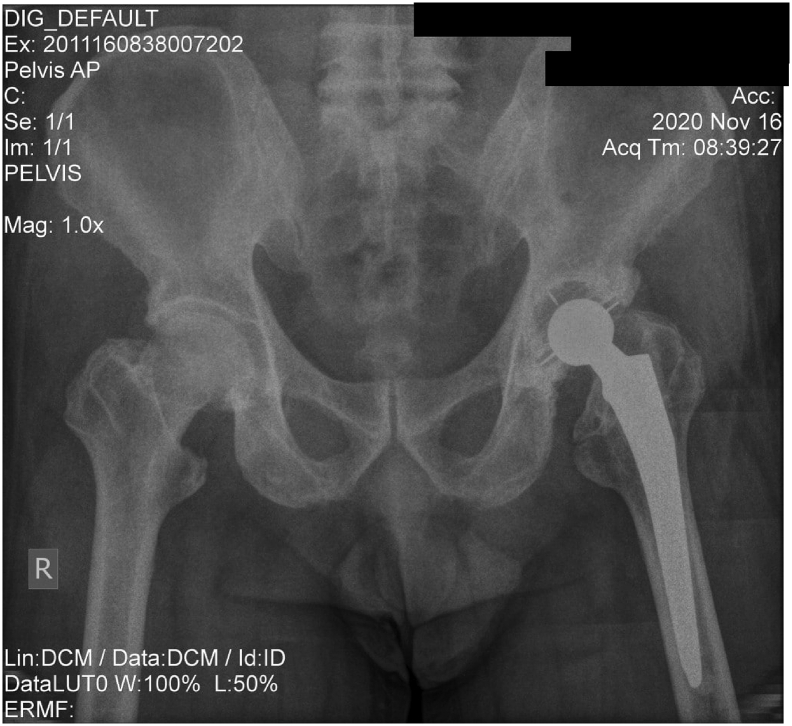
Current x-ray.

5-years follow-up in the clinic after second operation the patient's condition was very satisfactory. He could carry out normal daily activity with some limitation on hip flexion. Harris hip scoring was measured and showed 95 out of 100 which means excellent (Fig. 3). There was no leg length discrepancy, and patient could squat with slight limitation of 80 degrees hip flexion, normal abduction, adduction, external rotation and internal rotation (Fig. 4, Fig. 5). Radiologic examination showed no sign of loosening of the implant (Fig. 6).

## 3 Discussion

The Girdlestone procedure is indicated when the infection cannot be adequately controlled; soft tissue coverage as well as bone quality is not strong enough for new prosthesis; and multiple comorbidities that cause patients are unfit for surgery [2]. There are several reports about Girdlestone procedure result that analyze one or more following aspects such as healing of infection, leg length discrepancy, residual pain, Trendelenburg gait, oxygen consumption, walking limitations and external supports necessary, functional limitation and overall patient satisfaction [4].

There is a general agreement that resection arthroplasty is a direct method of healing infection. Some reports showed variably results from 80%–90% of healing rate or more optimistic result such as 90% to 100% of healing rate. In other hand bad result reported in rheumatoid arthritis (61%), enterococcal infection (76%), and methicillin-resistant *Staphylococci* (84%) [4].

Classically, Girdlestone situation is a painless situation, but according to literature, in some cases, pain is remained. Residual severe pain is reported in 16% to 33% of patients, moderate pain in 24% to 53% and mild pain in 76% of patients. Although this residual pain occurs in some report, this procedure has satisfactory pain relief in 85% to 100% of patients. For many years, it has been known that resection arthroplasty suffers a greater oxygen consumption 264% than normal [4].

Girdlestone situation, obviously greatly limits patient's walking ability. This condition more bother in elderly patients, which in significant proportion is up to 45% are unable to walk and 29% walk with walking aid either one or two crutches. Beside walking limitation, Girdlestone situation also provide global functional limitation. Universally validated hip scales, Harris Hip Scores revealed severe impairment in this condition. The scores range from 25 to 39, 51, 53 in methicillin-resistant cases. Patient's satisfactory with this procedure is vary widely and is completely subjective evaluation. Some literatures offer low percentages in 13%, another one is 59% to 74% and the best result is 83% of satisfied patients [4], [6].

Considering based on literatures, indications for Girdlestone procedure are non-ambulatory patients, immunocompromise, dementia, drug abuse, and impossible implantation. In this case, hip the choice of resection arthroplasty that not followed directly by implantation of prosthesis is based on doubt infection control. In our case, the patient fulfilled the last indication, in which the patient previously refused subsequent total hip arthroplasty as the treatment of choice in hip infection. Immediate pain relief and cost benefit on patient's perspective are other consideration to not directly did total hip replacement. As also mentioned in study conducted by Cordero-Ampuero et al., Girdlestone situation may be chosen in which the patient refused for implantation. Eradication of infection is the major purpose in this case. Fortunately, this situation quite satisfying the patient. Pain was diminished and he could walk with a cane after nine months postoperatively [4], [7]. This is similar to another case series conducted by Oheim et al. in which 23 patients (26 hips) with hip-joint empyema showed significant pain relief. This study further concluded that resection arthroplasty introduced by Girdlestone still a treatment of choice to control infection despite its poor functional outcome [8].

As previously stated, Girdlestone procedure is also widely used in prosthetic joint infection (PJI). Previous study from Castellanos et al. reported satisfactory pain relief in 83% of patients treated with Girdlestone procedure [9]. Similar result was also shown in study conducted by Grauer et al., in which pain was alleviated in all patients after Girdlestone procedure was performed. However, it needs to be highlighted that Girdlestone procedure result in poor functional outcome, therefore resection of the infected joint should spare as much of proximal end of the femur for future possibility of hip replacement [10].

Conversion of Girdlestone situation to a total hip replacement is a challenging procedure that may face difficulty in the absence of abductor mechanism with major acetabular deficiency. This procedure considered as one of the greatest challenges in adult reconstructive surgery. Comprehensive and complete preoperative evaluation demanded to achieve good result of this technique. Some indications of THR conversion following Girdlestone procedure are walking difficulties, limb length inequality, pain, persistent infection, poor medical condition, high risk of recurrent infection, unreconstructable bone stock deficiency, abductor mechanism problem and neuromuscular disorder [11]. In this case, walking difficulties and limb length inequality were the major reason to perform conversion.

Another alternative management in PJI include cement spacer implantation. A study conducted by Bialecki et al. further concluded that prolonged use of spacers has been associated with spacer dislocation, bone stock defect progression, fractures, and possibility of developing bacterial resistance. Therefore, Girdlestone procedure is widely accepted as the treatment of choice in cases with inadequate bone stock and possible difficulty to treat infection, similar to the condition in our patient [12].

Use of specific prosthesis may also affect surgical outcome in the patient. Dual mobility bearing system has been introduced early to provide increased stability in THR. Biomechanical studies also showed better stability using dual mobility prosthesis with further reduction in dislocation rates. However, adverse soft-tissue reactions needed to be taken into consideration while choosing dual mobility prosthesis particularly in cementless shells [13].

In our patient, two-stage surgery consisted of Girdlestone procedure followed by THR resulted in excellent functional outcome after 5-year follow-up. This may be a treatment of choice in which the patient previously refused prosthetic implantation or there was uncontrolled joint infection. Girdlestone procedure is an essential surgery to eradicate joint infection and the procedure itself result in significant pain alleviation. However, due to the functional limitation that ensues, THR is indicated to correct walking difficulties and limb length discrepancy after resection from Girdlestone procedure.

## 4 Conclusion

In conclusion, Resection arthroplasty of the hip followed by a conversion to total hip replacement in hip infection case provide complete infection eradication, good functional outcome and satisfaction for the patient. Comprehensive preoperative evaluation is a mandatory Although the procedure was time-consuming, technical demanding, high risk for poor functional outcome it can be a choice if the eradication of infection still in a doubt and other surgical requirements within some limitations.

## Source of funding

The authors report no external source of funding during the writing of this article.

## Ethical approval

Ethical approval was not required in the treatment of the patient in this report.

## Consent

Written informed consent was obtained from the patient for publication of this case report and accompanying images.

## Registration of research studies

Does not need any registration.

## Guarantor

Guntur Utama Putera, MD

## Provenance and peer review

Not commissioned, externally peer-reviewed.

## CRediT authorship contribution statement

Guntur Utama Putera contributes to the study concept or design, data collection, analysis, interpretation and writing the paper.

Iman Solichin contributes to the study concept, oversight and leadership responsibility for the research activity planning and execution including mentorship external to the core team.

Mohamad Walid Kuncoro contributes to the study concept or design, data collection and writing the paper.

## Declaration of competing interest

The authors declare no conflicts of interest.
